# Efficient Removal of Methylene Blue Using Living Biomass of the Microalga *Chlamydomonas moewusii*: Kinetics and Equilibrium Studies

**DOI:** 10.3390/ijerph19052653

**Published:** 2022-02-24

**Authors:** Raquel Seoane, Sergio Santaeufemia, Julio Abalde, Enrique Torres

**Affiliations:** Laboratorio de Microbiología, Facultad de Ciencias, Campus de A Zapateira, Universidade da Coruña, 15071 A Coruña, Spain; raquel.seoanec@gmail.com (R.S.); sergio.santaeufemia.sanchez@udc.es (S.S.); abaldej@udc.es (J.A.)

**Keywords:** microalga, dye, biosorption, bioremediation, decolorization, environmental pollutants

## Abstract

The efficiency of the living biomass of the microalga *Chlamydomonas moewusii* in removing methylene blue dye is determined. The kinetics, equilibrium isotherms, and the effects on this process of the pH, contact time, and initial concentration of the dye are studied. Fourier transform infrared spectrometry and point of zero charge are used to characterize the biomass and explore the process. The maximum removal capacity derived from the Langmuir isotherm is 212.41 ± 4.55 mg/g after 7 h of contact time at pH 7. The removal process is rapid because kinetic studies revealed that the best fit of the data is with pseudo-third-order kinetics. The removal efficiency is dependent on the pH; as the pH increased, the efficiency is higher. These results show that the living biomass of this microalga is a very efficient biosorbent and therefore very suitable for the removal of methylene blue from aqueous solutions.

## 1. Introduction

Within the great variety of pollutants present in water, dyes constitute one of the largest and most important groups. They are compounds of common use in the industry, highlighting textiles as the main emitter, although they are also used in the paper, food, medical, etc. industries [1]. Because of this activity, the dyes end up, along with the rest of the pollutants in the wastewater, in the aquatic ecosystems. In these ecosystems, the presence of dyes is very apparent since only small amounts (usually less than 1 mg/L) are needed to be visible. They absorb and reflect sunlight, interfering with its proper penetration into the water column, altering the solubility of gases, and therefore seriously disturbing aquatic life and the food chain. In recent decades, knowledge about the toxicity of the most commonly used organic dyes has increased; some of these are now considered carcinogenic or mutagens, and for this reason, their elimination from water has become a priority environmental health issue [2,3,4].

Methylene blue is one of the most widely used dyes; it is a cationic dye used in the coloring of paper, wool, and cotton; it is useful in microbiology, surgery, clinics, and diagnosis [5]. Akin to other dyes, methylene blue has serious environmental repercussions, and although its toxicity is not considered too high (a safe drug when used in therapeutic doses), it can cause vomiting, increased heart rate, confusion, and tissue necrosis with prolonged exposure. Akin to the rest of the organic dyes of a synthetic nature, methylene blue is considered a recalcitrant compound, resistant to aerobic degradation and stable against oxidizing agents, resulting in a very low natural elimination. For this reason, effective and inexpensive methods are needed for its elimination.

Conventional treatment processes such as advanced oxidative processes, precipitation, or membrane separation have disadvantages such as their high cost, poor performance, or generation of waste. Adsorption processes have been an excellent alternative. Compared to traditional sorbent materials, such as activated carbon, new materials have been proposed (natural materials, industrial and/or agricultural waste, microbial biomass...) that are more economical, efficient, and respectful with the environment. Since these materials have a biological origin, the technology that uses them is called biosorption. Biosorption has been used with varying degrees of success for the removal of methylene blue from aqueous solutions [6,7,8]. Additionally, although many materials have been evaluated for this purpose, the search for suitable materials is a fundamental pillar for the development of this technology. In this sense, microalgal biomass is considered an excellent alternative to be used in this technology. This biomass can be used alive or dead [9], with little or no modification [10], it can be efficiently immobilized on various matrices [11] and used in biotechnological processes (both immobilized and free), its production is low-cost, and at the same time, other benefits such as CO_2_ fixation [12] or the production of biofuels [13] can be obtained. The evaluation of this type of biomass to be used efficiently in biosorption processes is a current topic.

The use of living biomass is not very widespread and has been little evaluated in biosorption processes [14,15,16,17]. However, the use of living cells can offer interesting advantages. Since cells are active, bioaccumulation and biotransformation can increase the amount of pollutants removed, increasing performance. In addition, only production and harvest are necessary to use the living biomass in these processes, since no subsequent treatments are necessary. More studies using living biomass as biosorbents to remove pollutants are required to adequately assess this strategy.

The aim of this work is to study the properties of the living biomass of the microalga *Chlamydomonas moewusii* to act as a low-cost sorbent without transformation in the removal of methylene blue from an aqueous solution. In general, the species of the genus *Chlamydomonas* are easy to produce and have been widely studied, for this reason, it is interesting to test this biomass for its application to the elimination of pollutants.

## 2. Materials and Methods

### 2.1. Obtaining the Microalgal Biomass

For the present study, the CCAP 11/5B strain of *Chlamydomonas moewusii* Gerloff obtained from the Culture Collection of Algae and Protozoa (CCAP) of the Dunstaffnage Marine Laboratory (Scotland, United Kingdom) was used. The medium used for the culture of this microalga was Bristol sterilized in an autoclave at 120 °C for 20 min. The biomass of this microalga was obtained from stock cultures in the laboratory. The cultures were carried out in 1 L Pyrex bottles and incubated in a culture chamber at a controlled temperature of 18 ± 2 °C. The cultures were aerated using atmospheric air filtered through Millipore filters of 0.22 µm pore size and with a constant flow of 10 L/min. Illumination was 68 μE/(m^2^ s) with a 12:12 h light-dark cycle.

### 2.2. Stock Solution of the Dye

Three stock solutions of the dye were prepared to allow an adequate volume to be added into the tubes of the experiments to obtain the final concentration of the dye necessary for each test. The stock concentrations used were 100, 1000, and 5000 mg/L. Each stock concentration was obtained by dissolving a suitable amount of the dye powder in deionized water.

### 2.3. Characterization of the Biosorbent

Fourier transform infrared spectroscopy (FTIR) and the point of zero charge (pH_PZC_) were used to characterize the *C. moewusii* biomass.

#### 2.3.1. Fourier Transform Infrared Spectroscopy (FTIR) Analysis

FTIR was used to recognize functional groups on the biomass before and after the biosorption of methylene blue. The spectra were obtained by applying the attenuated total reflection (ATR) mode on the FTIR spectrometer (Thermo Scientific Nicolet iS10). The specifications were the following: 64 cumulative scans, a range of 525–4000 1/cm and resolution spectrum of 4 1/cm. The biomass was obtained by centrifugation at 4500 *g* for 15 min and was previously dried and ground before these analyses.

#### 2.3.2. Point of Zero Charge (pH_PZC_) Determination

The pH drift test was used for the determination of the point of zero charge. Six pH solutions (2–13) with 50 mL of 0.1 M NaCl were used in the test. The pH of each solution was adjusted to these initial pHs using HCl or NaOH. In total, 40 mg of microalgal biomass was added to each of the pH solutions. This 40 mg of biomass was obtained as follows: a volume of the microalga stock culture enough to obtain a number of cells equivalent to 40 mg of dry weight (Neubauer chamber counting was used) was centrifuged at 4500 *g* for 15 min. The cells obtained were washed in the 0.1 M NaCl solution. After a new centrifugation, the collected biomass was resuspended in each pH solution. The biomass in these solutions was stirred for 3 h, and the final pH was recorded.

### 2.4. Batch Biosorption Studies

The experiments were carried out in 50 mL Kimax tubes with a duration of 7 h in which the cells were exposed to different concentrations of methylene blue. The concentrations tested were the following: 0.75, 2.25, 4.5, 9, 12, 24, 48, 96, 200, and 400 mg/L.

To each tube the following was added:

- A determined volume of the stock culture of the microalga equivalent to an amount of dry biomass of 40 mg (0.8 g/L) (this volume was also obtained after determining the cell density of the culture by means of a Neubauer chamber);
- An adequate volume of any methylene blue stock solution according to the concentration tested;
- Sterile deionized water until reaching a final volume of 50 mL.

The tubes were shaken at 200 rpm by means of an orbital shaker. The final pH was kept around 7.0 ± 0.4 at all concentrations tested. To keep the cells active, the tubes were incubated in the culture chamber at 18 ± 2 °C with a light intensity of 68 μE/(m^2^ s). Control tubes exposed to light and in the dark (covered with aluminum foil) both without cells were included in the experiments with the purpose of studying the stability of the dye in darkness and light (photodegradation). All experiments were performed in triplicate.

Aliquots were taken from each tube to determine the amount of methylene blue at 0, 0.042, 0.08, 0.25, 0.5, 1, 1.5, 2, 3, 5, 6, and 7 h. These aliquots were centrifuged at 13,000 *g* for 10 min at 4 °C. The supernatant was transferred to an Eppendorf tube and stored at −20 °C until quantification of methylene blue.

### 2.5. Determination of the Effect of pH

Several pHs (2–10) were tested to investigate the effect of pH on the removal of methylene blue by this microalgal biomass for 7 h and at a dye concentration of 200 mg/L. Control tubes with dye but without biomass, both in light and in darkness, at the same pHs were also included in these experiments to detect possible variations in the concentration of the dye not due to the effect of biomass. The conditions of the experiments were the same as those indicated above. The pH was measured with a Basic 20 pH meter (Crison). Sodium hydroxide or hydrochloric acid was used to adjust the pH of the solutions.

### 2.6. Analytical Method

The methylene blue concentration in the supernatants was determined by measuring its absorbance at 665 nm using a Shimadzu UV-1700 spectrophotometer (Japan). Previously, a calibration curve was made with solutions of known methylene blue concentration to obtain the equation of a line that allowed calculating the concentration of the dye as a function of the absorbance measured.

The amount of dye removed by the biomass was calculated by the following equation:

(1) $$q_{t}=\left(\left(C_{i}-C_{l}\right)-C_{t}\right)*V/m$$

where *q_t_* is the amount of methylene blue removed per unit of biomass (mg/g) at time *t* (h), *C_i_* is the initial concentration of the dye, *C_l_* is the concentration of the dye at time *t* in the control tube exposed to light but without cells, *C_t_* is the concentration of the dye at time *t* in the supernatant of the tubes with the cells (all concentrations in mg/L), *V* is the volume of the solution (L) and *m* the total mass of microalgae (g).

### 2.7. Removal Kinetics Analyses

Five kinetic models were evaluated to establish which model best describes the removal kinetics of this dye by the living biomass of the microalga. These models ranged from pseudo-first-order to pseudo-fourth-order. An intraparticle diffusion model was used to understand a possible mechanism. Their equations are shown in Table 1.

### 2.8. Isotherm Studies

Sorption isotherms were used to study the equilibrium characteristics of the sorption of this dye by the living biomass of the microalga. The isotherm models selected in this study are shown in Table 2.

### 2.9. Statistical Analysis

Data are represented as mean ± standard error of three independent experiments. A Student’s *t* test (α < 0.05) was used to compare the results obtained between two groups, and an ANOVA with Tukey’s test to compare between more than two groups (normality and homogeneity of variances were previously verified). Non-linear regression was used to fit the removal data to the equations of the different models (kinetic and isotherm) using SigmaPlot version 12.5 for Windows (Systat Software, Inc., Redmond, WA, USA). The error function initially selected to minimize the non-linear regression was the sum of the squares of the errors (*SSE*) given by the following formula:

(2) $$SSE=\sum_{i=1}^{n}{\left(V_{exp}-V_{calc}\right)}^{2}$$

where *V_exp_* is the experimental value obtained, *V_calc_* is the calculated value from the model, and *n* is the number of observations in the experiment.

To establish the goodness of fit and choose the model that best explains the data, the regression coefficient (*r*^2^) and the Akaike information criterion (AIC) were used.

## 3. Results and Discussion

### 3.1. Stability of the Dye: Photodegradation

To determine the stability of methylene blue, and thus be able to attribute its removal only to the effect of biomass, experiments were carried out with the same conditions as those for biosorption but without adding biomass. The tubes with the dye exposed to light allowed us to study if the dye underwent photodegradation during the time that the experiments lasted. While the tubes in the dark allowed us to determine losses of the dye due to other possible factors. Comparison by means of a Student’s *t* test (paired samples) of the initial concentration of dye (at time zero) with the concentration of dye after 7 h of exposure to the intensity of light used for the culture of the microalga, indicated that there were no significant differences (*t*_29_ = −1.53, *p* = 0.137) in the concentration of dye. A photodegradation effect of methylene blue was ruled out of all concentrations tested. A similar result was obtained with the tubes in the dark (*t*_29_ = −1.21, *p* = 0.237). Therefore, this dye remained stable during the experiments and with the exposure conditions tested. This result coincides with that obtained in biosorption experiments with the microalga *Phaeodactylum tricornutum* under culture conditions (illumination) similar to those used in these experiments. These results show the stability of some dyes, such as methylene blue and crystal violet, under standard microalgae culture conditions. However, unlike these, there are also other dyes, such as safranin, that are susceptible to photodegradation under these conditions [18]. For this reason, it is important to verify this process when using living microalgal biomass under culture conditions. This result of no photodegradation of methylene blue under usual culture conditions agrees with the fact that aggressive conditions (high degree of oxidation and light intensity) are necessary for the physicochemical degradation of methylene blue. To achieve these conditions, certain reagents are necessary that can increase the cost of the process and can be very disrespectful with the environment [19,20]. For this reason, having an efficient biological removal process would be an excellent alternative.

### 3.2. Fourier Transform Infrared Spectroscopy (FTIR) Analysis

**Figure 1:**
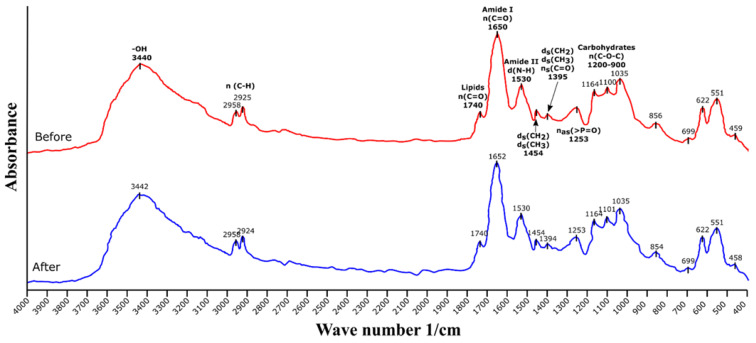
FTIR spectra of *Chlamydomonas moewusii* before and after the removal process of methylene blue.

Figure 1 shows the FTIR spectra of the biomass of *C. moewusii* before and after the methylene blue removal process. In the spectra, the three main characteristic regions of this type of spectra with microalgae can be observed. The peak around 1740 1/cm corresponds to the lipids; the regions between 1600–1690 1/cm and 1480–1575 1/cm are called amide I and amide II, respectively, and correspond to amide groups associated with proteins; finally, the region 900–1200 1/cm corresponds to carbohydrates. The broad peak at 3440 1/cm corresponds to the presence of -OH groups, the peaks at 2925 and 2958 1/cm correspond to the stretching of the C-H bonds, the peaks from 1395–1460 1/cm corresponds to the bending vibration of CH_2_ and CH_3_ groups, and symmetrical stretching of the C=O group in the formation of carboxyl (-COOH). The peak around 1253 1/cm corresponds to organic phosphates [21,22].

No significant changes in the spectra were observed before and after the removal process, which seems to indicate that the main mechanism of removal was by ion exchange between the negatively charged groups of the microalgal cells and the cationic dye. This is a common result in methylene blue biosorption processes using biomass. That is, FTIR spectra are similar before and after sorption or have slight shifts in the peaks corresponding to some functional groups (amine, hydroxyl, C=O, and C-O) [23,24,25]. This indicates that electrostatic attraction is the main biosorption mechanism for this compound. The dye binds electrostatically to certain components of the biomass. In the case of microalgal biomass, there are many components, mainly located on the cell surface, that contribute to this process because they provide negative charges (negative zeta potential predominately caused by dissociated carboxylic groups) that facilitate the union of cationic dyes [26]. This may be one of the reasons why microalgal biomass is very suitable for biosorption of cationic sorbates.

### 3.3. Point of Zero Charge (pH_PZC_)

**Figure 2:**
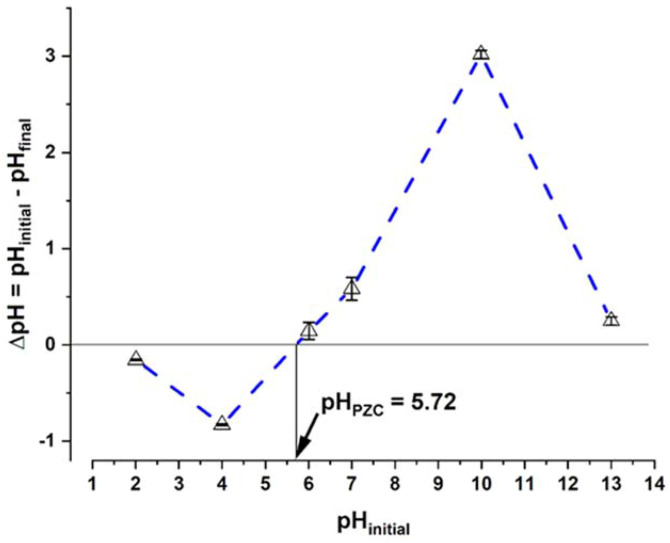
Point of zero charge (pH_PZC_) determination of *C. moewusii* biomass.

The sorption processes of ionic compounds are highly influenced by the pH [27]; therefore, the determination of the sorbent’s pH_pzc_ is important, since, depending on this value, the sorption may or may not be favored. The surface of algae has various functional groups whose charges can vary with pH. When the charge of these groups is opposite to that of the sorbate, sorption is favored. In the case of the living biomass of *C. moewusii*, the pH_pzc_ obtained was 5.72 (Figure 2). Above this pH, the biomass of this microalga will be negatively charged.

### 3.4. Effect of Contact Time on the Removal of Methylene Blue by Living Cells of the Microalga

**Figure 3:**
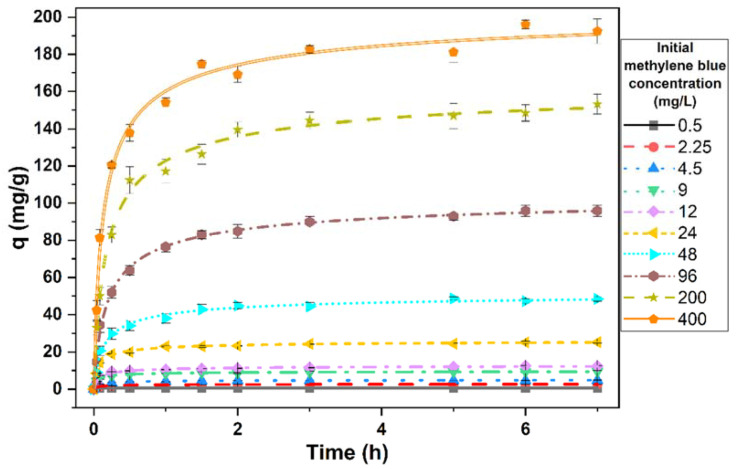
Evolution of the amount of methylene blue removed per unit of biomass throughout the contact time. Points represent the means of three replicates and bars indicate the standard deviation.

Figure 3 represents the amount of dye removed per unit of biomass during the time. The amount of dye removed after 7 h was statistically significant (*t_29_* = 4.46, *p* < 0.0001). This removal was due to the effect of biomass, since, as previously demonstrated, the amount of dye did not vary in the absence of biomass. This removed amount increased quickly at the start of the process. The speed was faster in the lower concentrations of methylene blue and decreased as the concentration increased. After this initial phase, an equilibrium was observed. Only about 0.5 h was needed to reach this equilibrium at the lowest concentration of the methylene blue tested. This time increased to 1.5 h at the highest concentration. The abundance of free sites in the biomass would explain this initial speed, which decreases as they become saturated with the dye.

### 3.5. Effect of the Initial Dye Concentration

The initial concentration of methylene blue had a significant effect on the amount of dye removed (ANOVA, *F*_9,20_ = 6190.31, *p* < 0.0001) (Figure 3). According to this figure, an increase in the initial dye concentration caused an increase in the concentration of dye eliminated. With an initial dye concentration of 0.5 mg/L, the amount of dye removed per unit of biomass was 0.64 ± 0.03 mg/g, whereas with the highest concentration tested (400 mg/L), the amount eliminated was 192.71 ± 8.92 mg/g.

However, when the removed amount of dye was expressed as a percentage of the added initial amount (Table 3), the percentage decreased when the initial concentration of dye increased. At the lowest concentration tested, almost all the added methylene blue was removed; however, this percentage progressively decreased to 38.7 ± 1.3% at the highest initial concentration. As the initial dye concentration increases, there are more residual dye molecules that do not have access to the sorption sites and therefore remain in the solution. This behavior is common in these types of processes [28,29].

### 3.6. Removal Kinetics

The kinetic studies allow the characterizing of the removal process and deducing of a possible mechanism. For these reasons, it is important to fit the experimental data to the best possible model. In this study, five kinetic models (Table 1) were evaluated in order to find the model that best explains the removal of methylene blue using living cells of this microalga. Although the pseudo-first-order and pseudo-second-order models are the most used [27,30], two additional models have been evaluated in this work (pseudo-third and pseudo-fourth order) due to the high speed observed in the removal. The adjustment of the experimental data to these models confirmed that the model that best defined the obtained results was the pseudo-third-order. This model is the one that obtained the highest *r*^2^ values and the lowest values of the AIC in all the methylene blue concentrations tested (Table 4).

The kinetic parameters obtained with this model are shown in Table 5. With these parameters and considering this model, the half-removal time (*t*_1/2_, the time required for the removal to reach half of the equilibrium value) can be calculated using the following formula:

(3) t1/2=1(2∗qe2∗k3)

As can be seen in Table 5, the half-removal time was very short at all concentrations tested. This time it increased with the initial concentration of methylene blue. However, it only took 3.6 min at the highest methylene blue concentration. This shows the high affinity of this dye for the biomass of this microalga.

The intraparticle diffusion model was used to study the mechanism and the limiting step in the removal process of this dye by the living microalgal biomass. Although this model was the worst adjusted to the data obtained (Table 4); therefore, intraparticle diffusion does not appear to be an important mechanism in this process, the information that this model provided was interesting to study the process. The parameters obtained with this model are shown in Table 6. Since the value of the intercept (*I*) is not zero and is a high positive value, there is a rapid initial removal in a short period of time. This is consistent with the fit obtained for pseudo-third-order kinetics. Furthermore, this is indicative that intraparticle diffusion is not the only step that controls the rate of the process.

**Figure 4:**
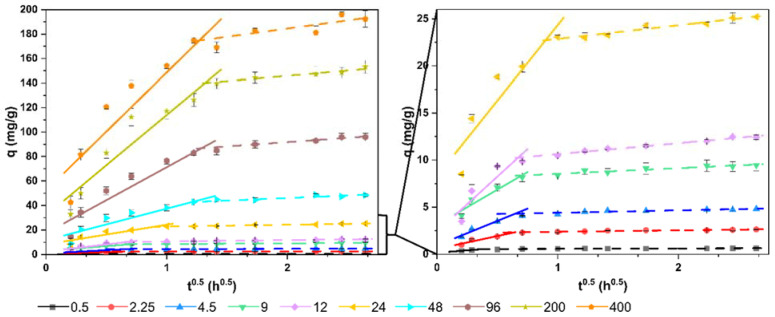
Linear plots of the intraparticle diffusion kinetic model.

In fact, the linear representation of this model (*q* vs *t*^0.5^) (Figure 4) showed multilinearity in all the concentrations tested. This indicates that there are several mechanisms controlling the process. Two steps can be well differentiated. An initial step of rapid removal, mainly due to sorption to the surface of the cells, and a second, more gradual step, until equilibrium is reached. This step had a higher speed (greater slope of the line) as the concentration of the dye increased (from 0.11 in the concentration of 0.5 mg/L to 13.40 in the concentration of 400 mg/L). In this second step, the entry of the dye into the cells could have a greater role.

### 3.7. Isotherm Studies

**Figure 5:**
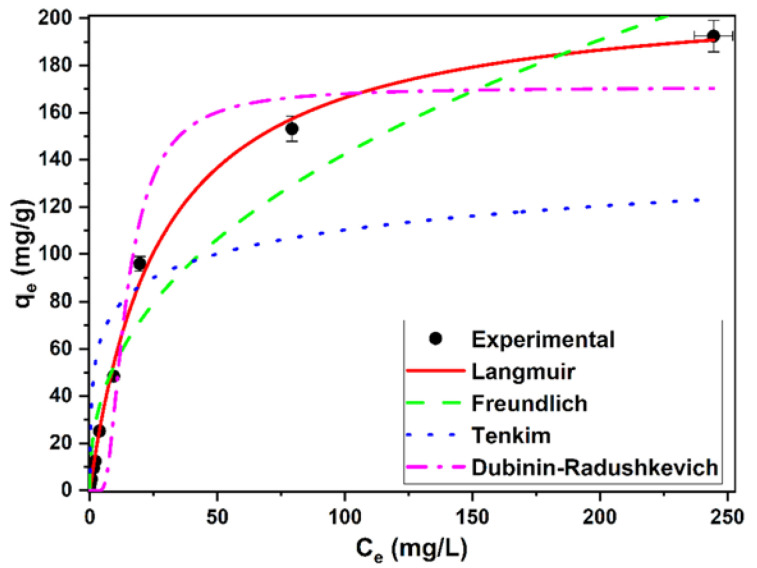
Equilibrium isotherms for methylene blue removal using living biomass of the microalga *C. moewusii*.

Figure 5 shows the fit of the data to the isotherm models. The parameters derived from these models are shown in Table 7. Considering the values obtained from the correlation coefficients (*r*^2^) and the AIC, the data were better fitted with the Langmuir model. Therefore, this model provided a better description of the experimental data. From this model, the value of the maximum removal capacity of methylene blue was 212.41 ± 4.55 mg/g. In this study, the separation factor values (*R_L_*, Table 1), derived from the Langmuir model and calculated for all the methylene blue concentrations tested were 0.98, for the lowest initial concentration of the dye, and 0.06 for the highest concentration. Since there were values greater than zero and less than one, the removal of the dye by this biomass can be considered favorable in all the concentrations tested [31].

The Freundlich model also provides interesting information about the process. The constant *K_F_* is related to the affinity for sorbate. The value obtained with this biomass can be considered quite high (20.41 *mg^1−(1/n)^ L^1/n^/g)*, implying a high affinity. In addition, the parameter *1/n*, which is related to the heterogeneity of the sorbent, obtained an intermediate value between 0.2 and 0.8, indicating a moderate heterogeneity of this biomass. Temkin’s model obtained the worst fit of the data. This model can be used to calculate the heat of the process. The constant *b_T_* was positive, which indicates that the removal process was exothermic. Finally, the Dubinin–Radushkevich model is used to calculate the mean free energy and to interpret the mechanism involved. If this value is between 8 and 16 kJ/mol, the process would be more related to chemosorption, while a value below 8 kJ/mol would indicate a physical process. The value obtained with this biomass was very low, which would indicate that the methylene blue sorption mechanism would be physical. This would be in agreement with the results of the analysis of the FTIR spectra indicated above.

### 3.8. Effect of pH

The initial pH of the solution is one of the main factors affecting the efficiency of an ionic compound sorption process [27]. The pH alters the degree of ionization of the sorbate and, at the same time, it affects the surface charge of the sorbent because it alters the ionization of different functional groups that the sorbent may have. In this sense, it would be desirable that the charges of the sorbate and sorbent be opposite to favor electrostatic interaction. Previously, it was also verified with the tubes exposed to light and in darkness that the dye remained stable at the pHs tested (2–10). Both the dark (*t*_17_ = −0.021, *p* = 0.984) and light (*t*_17_ = 1.363, *p* = 0.191) tubes did not show variations in the initial concentration of the dye after 7 h at the tested pHs.

**Figure 6:**
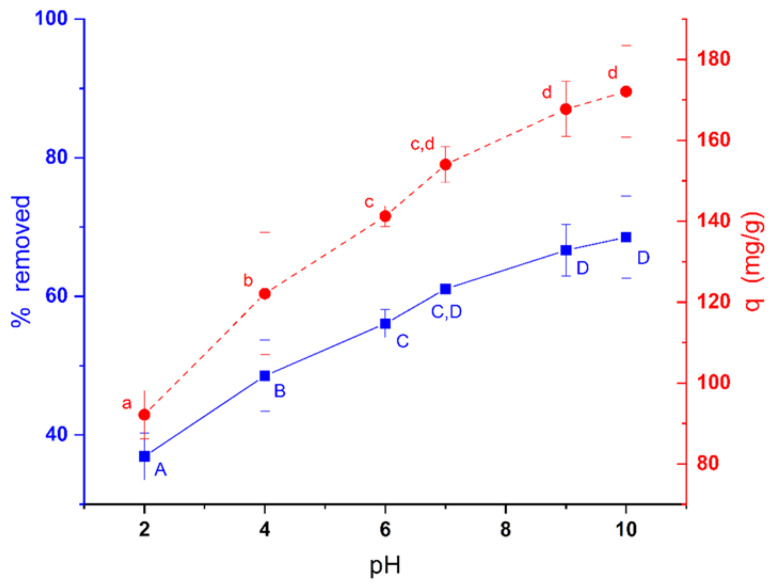
Effect of pH on the efficiency of methylene blue removal using 200 mg/L of dye. Different letters indicate significant differences in the Tukey test (α = 0.05).

Figure 6 shows the effect of pH on the efficiency of methylene blue removal by *C. moewusii* biomass using an initial methylene blue concentration of 200 mg/L. As can be seen in the figure, the efficiency of dye removal increased with increasing pH. This increase was statistically significant both for the percentage removed (ANOVA, *F*_5,12_ = 74.491, *p* < 0.0001) and for the amount of dye removed per unit of biomass (ANOVA, *F*_5,12_ = 84.734, *p* < 0.0001). The lowest removal capacity was at pH 2 with 92.14 mg/g (36.9%). Since the zero-charge point of this biomass was 5.72, at pHs lower than this value, the surface charge would be positive, and since methylene blue is a basic dye, its charge would also be positive, which would cause repulsion. As the pH rises, the positive charge of the biomass decreases, and when the pH exceeds the value of the zero point, the charge will be negative, which facilitates the electrostatic interaction between the biomass and the positive charge of methylene blue (*p_ka_* = 3.14), increasing the removal. In fact, the removal capacity was 172.09 mg/g (68.5%) at pH 10, which represented an increase of 31.6% with respect to pH 2.

This electrostatic effect may not be the only one responsible for this variation observed with pH in the removal capacity of this biomass. Since living biomass is used, pHs far from the optimal pH for the culture will cause physiological alterations with negative effects on the metabolism of the cells. These negative effects can influence the removal process because they will alter the additional bioaccumulation and biotransformation reactions that live biomass can have under suitable culture conditions to favor the removal process. This effect could be more intense towards low pHs since many microalgae have optimum growth pHs in the neutral or slightly alkaline region, which implies that microalgae can better tolerate more extreme alkaline pHs [32,33]. In the alkaline pH range, the higher efficiency in the removal of methylene blue observed could be due to the joint action of the electrostatic effect and cellular metabolism.

### 3.9. Comparison with Other Sorbents

To assess the benefits of the living biomass of this microalga in the removal of methylene blue, it is interesting to compare this biomass with other sorbents already used for this purpose. Table 8 shows some examples collected from the literature. As can be seen, the biomass of this microalga presented a better performance than most of the used sorbents. Additionally, in many cases, it shows a great difference. It is noteworthy that some of the sorbents included in this Table have a high processing degree. Some sorbents require their transformation into carbon (biochar and hydrochar), which implies a high energy cost due to the use of high temperatures. Other sorbents are subjected to chemical transformations (exposure to acids, alkalis, oxidations) that consume reagents; or some sorbents are bound to new materials that may be expensive (magnetic particles, nanoparticles, or polymeric materials). The use of the living biomass of this microalga without transformation means less processing and, therefore, a lower cost. The only requirements are the culture and harvesting of the cells to obtain the biomass. The culture of microalgae is simple and offers many biotechnological possibilities [34]. Today, algal biomass is considered a relevant alternative, presenting advantages over terrestrial biomass such as higher growth rate, low water consumption, no competition for arable land, carbon-neutral emissions, and the production of numerous bioactive compounds [35]. Furthermore, biomass derived from microalgae is offering very good results in the fields of pollutant removal and wastewater treatment [18,36,37,38]. For these reasons, the biomass of this microalga can be very attractive for the elimination of this dye, considering the high efficiency shown.

The use of living biomass may have other advantages derived from the fact that it conserves metabolic activity. Due to this, the elimination of pollutants can be increased by bioaccumulation and biotransformation, which are added to the bioadsorption process. However, these two aforementioned processes are related to the possible toxic effect that the pollutant may have on the cells of that organism. For this reason, more resistant organisms will offer better possibilities. The species *C. moewusii* could be within this group. Although there are no data on the toxicity of this dye for this species, and no toxicity tests have been carried out in the present work, this tolerance would be a possible explanation for the high efficiency of this species, used as living biomass, in removing this dye. Taking this into account, it cannot be ruled out that these processes contributed to the good efficiency of the living biomass of this microalga, although they were not evaluated because the main objective of this work was to study the ability of this biomass to remove this dye. A similar case was the use of living biomass from the marine microalga *Phaeodactylum tricornutum*. This type of biomass was also more efficient than dead biomass in the elimination of safranin when the concentration of this dye was low [18]. Currently, there are more and more studies of pollutant removal using living microalgal biomass (or using cyanobacteria) [17,56,57], although they do not establish a comparison with dead biomass as in this work. In any case, these works demonstrate the good performance of this type of biomass and its potential as an eco-friendly and cost-effective sorbent.

## 4. Conclusions

The living biomass of the microalga *Chlamydomonas moewusii* is a suitable biosorbent for the removal of methylene blue. It is a biomass with a high affinity for this dye, is eco-friendly, and can be considered low-cost because it is a biomass that does not require transformation to achieve high performance; only culture is necessary, which allows this biomass to be easily available.

## Figures and Tables

**Table 1:** Kinetic models included in this study.

Kinetic Model	Differential Equation	Equation
Pseudo-first-order model	$\frac{dq}{dt}=k_{1}\left(q_{e}-q\right)$	$q=q_{e}(1-e^{-k_{1}t}$)
Pseudo-second-order model	$\frac{dq}{dt}=k_{2}{\left(q_{e}-q\right)}^{2}$	$q=\frac{q_{e}^{2}k_{2}t}{1+q_{e}k_{2}t}$
Pseudo-third-order model	$\frac{dq}{dt}=k_{3}{\left(q_{e}-q\right)}^{3}$	$q=q_{e}-\frac{q_{e}}{\sqrt[{2}]{\left(2*{q_{e}}^{2}*k_{3}*t+1\right)}}$
Pseudo-fourth-order model	$\frac{dq}{dt}=k_{4}{\left(q_{e}-q\right)}^{4}$	$q=q_{e}-\frac{q_{e}}{\sqrt[{3}]{\left(3*{q_{e}}^{3}*k_{4}*t+1\right)}}$
Intraparticle diffusion model (Weber–Morris)	-	$q=k_{i}t^{0.5}+I$

*q* (mg/g): mass of dye removed per unit of mass of biosorbent at time *t. q_e_* (mg/g): mass of dye removed per unit of mass of biosorbent at equilibrium. *k*_1_ (1/h), *k*_2_ (g/(mg h)), *k*_3_ (g^2^/(mg^2^ h)), *k*_4_ (g^3^/(mg^3^ h)), *k_i_* (mg/(g h^0.5^)), *k_S_* (1/h): constant rate of the respective model. *I* (mg/g): Intercept in the Weber–Morris model.

**Table 2:** Isotherm models included in this study.

Isotherm Model	Equations
Langmuir	$q_{e}=\left(q_{max}K_{L}C_{e}\right)/(1+K_{L}C_{e}$) $R_{L}=\frac{1}{1+K_{L}C_{0}}$
Freundlich	$q_{e}=K_{F}C_{e}^{1/n}$
Temkin	$q_{e}=\left(\frac{RT}{b_{T}}\right)Ln\left(A_{T}C_{e}\right)$
Dubinin–Radushkevich	$\;q_{e}=q_{max}e^{-B_{D}\varepsilon^{2}}$ $E_{D}=1/\sqrt[{2}]{2B_{D}}$

*C_e_* (mg/L): concentration of dye in solution at equilibrium. *q_e_* (mg/g): amount of dye removed at equilibrium per unit of mass. *q_max_* (mg/g): maximum removal capacity. *K_L_* (L/mg): constant related to removal capacity. *R_L_*: separation factor. *C_0_* (mg/L): initial sorbate concentration. *K_F_* (mg^1−(1/*n*)^ L^1/*n*^/g): constant related to the affinity for the biosorbent. *n*: intensity of removal. *A_T_* (L/mg): Temkin isotherm equilibrium binding constant. *b_T_* (g J/(mg mol)): constant related to the heat of removal. *T* (°K): absolute temperature. *R* (J/(mol K)): gas constant. *B_D_* (mol^2^/J^2^): free energy sorption per mole of the sorbate. *ε*: Polanyi potential calculated with the equation: $\varepsilon =RT\mathrm{Ln}(1+1/C_{e})$ *E_D_* (kJ/mol): apparent energy of removal.

**Table 3:** Percentage of methylene blue removed based on the initial concentration added.

	Initial Dye Concentration (mg/L)									
	0.5	2.25	4.5	9	12	24	48	96	200	400
*p* (%)	99.9 ± 0.2	92.4 ± 0.3	85.4 ± 0.6	83.8 ± 2.1	82.7 ± 0.9	84.2 ± 0.7	80.5 ± 1.2	79.6 ± 0.2	60.7 ± 0.7	38.7 ± 1.3

**Table 4:** Correlation coefficients (*r*^2^) and AIC (Akaike information criterion) values obtained for the data adjusted to the kinetic models.

	Kinetic Model									
Initial Methylene Blue Concentration (mg/L)	Pseudo-First-Order		Pseudo-Second-Order		Pseudo-Third-Order		Pseudo-Fourth-Order		Intraparticle Diffusion	
	*r* ^2^	AIC	*r* ^2^	AIC	*r* ^2^	AIC	*r* ^2^	AIC	*r* ^2^	AIC
0.5	0.965	−74.48	0.992	−93.29	0.996	−101.48	0.994	−95.63	0.421	−40.55
2.25	0.957	−35.97	0.992	−57.29	0.994	−60.14	0.989	−53.19	0.564	−8.16
4.5	0.955	−20.85	0.993	−44.91	0.997	−55.11	0.993	−44.86	0.590	5.88
9	0.953	−4.96	0.990	−24.39	0.996	−36.99	0.994	−31.58	0.557	22.03
12	0.954	2.419	0.983	−9.73	0.986	−12.11	0.983	−9.58	0.627	27.58
24	0.954	19.22	0.989	1.12	0.993	−3.30	0.989	1.613	0.600	45.36
48	0.940	39.94	0.983	24.42	0.991	17.60	0.989	18.28	0.727	58.11
96	0.955	53.51	0.991	34.20	0.996	23.69	0.995	25.66	0.767	73.26
200	0.953	64.47	0.989	46.41	0.995	37.60	0.994	39.19	0.755	84.48
400	0.941	72.34	0.985	55.98	0.993	47.55	0.992	48.16	0.721	91.15

**Table 5:** Kinetic parameters derived from the model that obtained the best fit to the data (pseudo-third-order).

	Kinetic Parameters (Pseudo-Third-Order)		
Initial Methylene Blue Concentration (mg/L)	*q_e_* (mg/g)	*k*_3_ (g^2^/(mg^2^ h^1^))	*t*_1/2_ (h)
0.5	0.66 ± 0.02	130.12 ± 12.85	0.009
2.25	2.80 ± 0.03	2.54 ± 0.27	0.025
4.5	5.14 ± 0.04	0.70 ± 0.05	0.027
9	9.90 ± 0.08	0.26 ± 0.02	0.020
12	13.11 ± 0.25	0.09 ± 0.01	0.033
24	26.81 ± 0.35	0.02 ± 0.003	0.028
48	53.31 ± 0.99	0.003 ± 4 × 10^−4^	0.066
96	108.07 ± 1.41	4.7 × 10^−4^ ± 4 × 10^−4^	0.092
200	169.35 ± 2.42	2.1 × 10^−4^ ± 2 × 10^−5^	0.082
400	210.10 ± 3.37	1.9 × 10^−4^ ± 2 × 10^−5^	0.060

**Table 6:** Parameters associated with the intraparticle diffusion model.

	Parameters	
Initial Methylene Blue Concentration (mg/L)	*k_i_* (mg/(g h^0.5^))	*I* (mg/g)
0.5	0.14 ±0.05	0.36 ± 0.07
2.25	0.69± 0.18	1.20 ± 0.26
4.5	1.30 ± 0.32	2.14 ± 0.47
9	2.41 ± 0.63	4.47 ± 0.93
12	3.48 ± 0.80	5.03 ± 1.17
24	6.86 ± 1.65	10.93 ± 2.45
48	15.48 ± 2.81	15.41 ± 4.17
96	32.28 ± 5.29	26.56 ± 7.84
200	49.88 ± 8.44	44.60 ± 12.51
400	60.37 ± 11.14	63.59 ± 16.51

**Table 7:** Values of the constants derived from the isotherm models used in this study, and the error functions (*r*^2^ and AIC) used to assess the goodness of fit.

Isotherm Model	Constants and Error Functions	Value
Langmuir	*q_max_* (mg/g)	212.41 ± 4.55
	*K_L_* (L/mg)	0.04 ± 0.002
	*R_L_*	0.06 − 0.98
	*r* ^2^	0.997
	*AIC*	35.60
Freundlich	*1/n*	0.42 ± 0.05
	*K_F_* (*mg^1−(1/n)^ L^1/n^ /g*)	20.41 ± 4.66
	*r* ^2^	0.950
	*AIC*	67.56
Temkin	*A_T_* (L/mg)	18.84 ± 28.61
	*b_T_* (g J/(mg mol))	165.53 ± 49.72
	*r* ^2^	0.531
	*AIC*	90.84
D–R	*q_max_* (mg/g)	170.75 ± 11.64
	*B_D_* (mol^2^/J^2^)	2.79 × 10^−5^ ± 7 × 10^−6^
	*E_D_* (kJ/mol)	0.13
	*r* ^2^	0.943
	*AIC*	67.61

**Table 8:** Comparison with other sorbents using for the removal of methylene blue.

Materials	*q_max_*^†^ (mg/g)	*K_F_*^††^ (mg^1−(1/n)^ L^1/n^/g)	Contact Time (h)	[Dye] (mg/L)	References
*Bifurcaria bifurcata*	2744.5	189.8	0.25	10–1000	[39]
*Fucus vesiculosus*	698.48	225.3	24	100–2500	[8]
Oil palm shell carbon	384.62	132.28	30	50–500	[40]
Brewer’s spent grain	298.35	69.51	7	5–250	[41]
Brazilian berry seeds (*Eugenia uniflora*)	189.6	34.4	3	25–200	[6]
Magnetic *Cortaderia selloana* flower spikes	119.05	1.41	0.5	25–350	[42]
Chestnut husk	117.2	19.4	0.67	50–500	[43]
*Chlamydomonas variabilis* activated by H_2_SO_4_	115	68.5	0.5	20–80	[21]
*Sargassum ilicifolium*	99.7	-	0.67	1.28–38	[44]
*Cyanthilium cinereum*	76.34	10.07	0.83	10–50	[7]
Clay	58.20	-	2	10–100	[45]
*Paspalum maritimum*	56.18	13.08	0.83	10–50	[7]
Wood apple rind carbon	40.1	21.3	2	10–100	[46]
Hydrogel P(AAm-co-AcA)	39.59	-	24	5–50	[47]
*Ipomoea carnea*	39.38	3.96	2.7	10–50	[23]
*Cystoseira barbatula*	38.61	81.8	6	5–100	[48]
Banana peel	20.80	1.34	24	10–120	[49]
Neem leaf powder	19.61	9.47	5	25–70	[50]
Orange peel	18.60	1.75	24	10–120	[49]
*Chlamydomonas variabilis* (dead biomass)	18.3	1.26	0.5	20–80	[21]
Coconut coir	15.59	0.98	2.33	60–100	[51]
*Ulva lactuca*	10.99	1.45	2	5–25	[52]
Spent rice biomass	8.3	-	2	25–50	[53]
Fly ash	5.57	4.38	2	20–60	[54]
Glass fibres	2.24	2.12	6	25–50	[55]
***C. moewussi* (living, unmodified)**	**212.41**	**20.41**	**7**	**0.5–400**	**This work**

**^†^** Parameter obtained from a Langmuir isotherm, **^††^** Freundlich constant: affinity measure for the sorbent.

## Data Availability

The datasets used or analyzed during the current study are available from the corresponding author on reasonable request.

## References

[1] Sabnis R.W. (2010). Handbook of Biological Dyes and Stains: Synthesis and Industrial Applications.

[2] Gita S., Shukla S.P., Deshmukhe G., Choudhury T.G., Saharan N., Singh A.K. (2021). Toxicity Evaluation of Six Textile Dyes on Growth, Metabolism and Elemental Composition (C, H, N, S) of Microalgae *Spirulina platensis*: The Environmental Consequences. Bull. Environ. Contam. Toxicol..

[3] Croce R., Cina F., Lombardo A., Crispeyn G., Cappelli C.I., Vian M., Maiorana S., Benfenati E., Baderna D. (2017). Aquatic toxicity of several textile dye formulations: Acute and chronic assays with *Daphnia magna* and *Raphidocelis subcapitata*. Ecotoxicol. Environ. Saf..

[4] Lellis B., Fávaro-Polonio C.Z., Pamphile J.A., Polonio J.C. (2019). Effects of textile dyes on health and the environment and bioremediation potential of living organisms. Biotechnol. Res. Innov..

[5] Oz M., Lorke D.E., Hasan M., Petroianu G.A. (2011). Cellular and molecular actions of Methylene Blue in the nervous system. Med. Res. Rev..

[6] Georgin J., Franco D.S.P., Netto M.S., Allasia D., Oliveira M.L.S., Dotto G.L. (2020). Treatment of water containing methylene by biosorption using Brazilian berry seeds (*Eugenia uniflora*). Environ. Sci. Pollut. Res. Int..

[7] Silva F., Nascimento L., Brito M., da Silva K., Paschoal W., Fujiyama R. (2019). Biosorption of Methylene Blue Dye Using Natural Biosorbents Made from Weeds. Materials.

[8] Lebron Y.A.R., Moreira V.R., de Souza Santos L.V. (2021). Biosorption of methylene blue and eriochrome black T onto the brown macroalgae *Fucus vesiculosus*: Equilibrium, kinetics, thermodynamics and optimization. Environ. Technol..

[9] Santaeufemia S., Abalde J., Torres E. (2019). Eco-friendly rapid removal of triclosan from seawater using biomass of a microalgal species: Kinetic and equilibrium studies. J. Hazard. Mater..

[10] Ma X., Yan X., Yao J., Zheng S., Wei Q. (2021). Feasibility and comparative analysis of cadmium biosorption by living *Scenedesmus obliquus* FACHB-12 biofilms. Chemosphere.

[11] Ahmad A., Bhat A.H., Buang A. (2019). Enhanced biosorption of transition metals by living *Chlorella vulgaris* immobilized in Ca-alginate beads. Environ. Technol..

[12] Chen C.Y., Chang H.W., Kao P.C., Pan J.L., Chang J.S. (2012). Biosorption of cadmium by CO_2_-fixing microalga *Scenedesmus obliquus* CNW-N. Bioresour. Technol..

[13] Bohutskyi P., Chow S., Ketter B., Fung Shek C., Yacar D., Tang Y., Zivojnovich M., Betenbaugh M.J., Bouwer E.J. (2016). Phytoremediation of agriculture runoff by filamentous algae poly-culture for biomethane production, and nutrient recovery for secondary cultivation of lipid generating microalgae. Bioresour. Technol..

[14] Santaeufemia S., Torres E., Mera R., Abalde J. (2016). Bioremediation of oxytetracycline in seawater by living and dead biomass of the microalga *Phaeodactylum tricornutum*. J. Hazard. Mater..

[15] Dil E.A., Ghaedi M., Ghezelbash G.R., Asfaram A. (2017). Multi-responses optimization of simultaneous biosorption of cationic dyes by live yeast *Yarrowia lipolytica* 70562 from binary solution: Application of first order derivative spectrophotometry. Ecotoxicol. Environ. Saf..

[16] Mohapatra R.K., Parhi P.K., Pandey S., Bindhani B.K., Thatoi H., Panda C.R. (2019). Active and passive biosorption of Pb(II) using live and dead biomass of marine bacterium *Bacillus xiamenensis* PbRPSD202: Kinetics and isotherm studies. J. Environ. Manag..

[17] Hifney A.F., Zien-Elabdeen A., Adam M.S., Gomaa M. (2021). Biosorption of ketoprofen and diclofenac by living cells of the green microalgae *Chlorella* sp.. Environ. Sci. Pollut. Res. Int..

[18] Santaeufemia S., Abalde J., Torres E. (2021). Efficient removal of dyes from seawater using as biosorbent the dead and living biomass of the microalga *Phaeodactylum tricornutum*: Equilibrium and kinetics studies. J. Appl. Phycol..

[19] Lyubimenko R., Busko D., Richards B.S., Schafer A.I., Turshatov A. (2019). Efficient Photocatalytic Removal of Methylene Blue Using a Metalloporphyrin-Poly(vinylidene fluoride) Hybrid Membrane in a Flow-Through Reactor. ACS Appl. Mater. Interfaces.

[20] Zhang D., Dai F., Zhang P., An Z., Zhao Y., Chen L. (2019). The photodegradation of methylene blue in water with PVDF/GO/ZnO composite membrane. Mater. Sci. Eng. C Mater. Biol. Appl..

[21] Moghazy M. (2019). Activated biomass of the green microalga *Chlamydomonas variabilis* as an efficient biosorbent to remove methylene blue dye from aqueous solutions. Water SA.

[22] Silva A., Coimbra R.N., Escapa C., Figueiredo S.A., Freitas O.M., Otero M. (2020). Green Microalgae *Scenedesmus obliquus* Utilization for the Adsorptive Removal of Nonsteroidal Anti-Inflammatory Drugs (NSAIDs) from Water Samples. Int. J. Environ. Res. Public Health.

[23] Mathivanan M., Rahman S.S.A., Vedachalam R., Kumar P.S., Sabareesh G., Karuppiah S. (2021). *Ipomoea carnea*: A novel biosorbent for the removal of methylene blue (MB) from aqueous dye solution: Kinetic, equilibrium and statistical approach. Int. J. Phytoremediation.

[24] Moghazy R.M., Labena A., Husien S. (2019). Eco-friendly complementary biosorption process of methylene blue using micro-sized dried biosorbents of two macro-algal species (*Ulva fasciata* and *Sargassum dentifolium*): Full factorial design, equilibrium, and kinetic studies. Int. J. Biol. Macromol..

[25] Liang J., Xia J., Long J. (2017). Biosorption of methylene blue by nonliving biomass of the brown macroalga *Sargassum hemiphyllum*. Water Sci. Technol..

[26] Ozkan A., Berberoglu H. (2013). Physico-chemical surface properties of microalgae. Colloids Surf. B Biointerfaces.

[27] Yagub M.T., Sen T.K., Afroze S., Ang H.M. (2014). Dye and its removal from aqueous solution by adsorption: A review. Adv. Colloid Interface Sci..

[28] Fathollahi A., Coupe S.J., El-Sheikh A.H., Sanudo-Fontaneda L.A. (2020). The biosorption of mercury by permeable pavement biofilms in stormwater attenuation. Sci. Total Environ..

[29] Kocaman S. (2020). Synthesis and cationic dye biosorption properties of a novel low-cost adsorbent: Coconut waste modified with acrylic and polyacrylic acids. Int. J. Phytoremediation.

[30] Michalak I., Chojnacka K., Witek-Krowiak A. (2013). State of the art for the biosorption process—A review. Appl. Biochem. Biotechnol..

[31] Hall K.R., Eagleton L.C., Acrivos A., Vermeulen T. (1966). Pore- and Solid-Diffusion Kinetics in Fixed-Bed Adsorption under Constant-Pattern Conditions. Ind. Eng. Chem. Fundam..

[32] Goldman J.C., Azov Y., Riley C.B., Dennett M.R. (1982). The effect of pH in intensive microalgal cultures. I. Biomass regulation. J. Exp. Mar. Biol. Ecol..

[33] Vadlamani A., Viamajala S., Pendyala B., Varanasi S. (2017). Cultivation of Microalgae at Extreme Alkaline pH Conditions: A Novel Approach for Biofuel Production. ACS Sustain. Chem. Eng..

[34] Raja R., Shanmugam H., Ganesan V., Carvalho I.S. (2014). Biomass from Microalgae: An Overview. J. Oceanogr. Mar. Res..

[35] Geada P., Moreira C., Silva M., Nunes R., Madureira L., Rocha C.M.R., Pereira R.N., Vicente A.A., Teixeira J.A. (2021). Algal proteins: Production strategies and nutritional and functional properties. Bioresour. Technol..

[36] Molinuevo-Salces B., Riaño B., Hernández D., García-González M.C., Alam M., Wang Z. (2019). Microalgae and Wastewater Treatment: Advantages and Disadvantages. Microalgae Biotechnology for Development of Biofuel and Wastewater Treatment.

[37] Torres E. (2020). Biosorption: A Review of the Latest Advances. Processes.

[38] Xiong J.Q., Kurade M.B., Jeon B.H. (2018). Can Microalgae Remove Pharmaceutical Contaminants from Water?. Trends Biotechnol..

[39] Bouzikri S., Ouasfi N., Benzidia N., Salhi A., Bakkas S., Khamliche L. (2020). Marine alga “*Bifurcaria bifurcata*”: Biosorption of Reactive Blue 19 and methylene blue from aqueous solutions. Environ. Sci. Pollut. Res. Int..

[40] Tan I.A.W., Hameed B., Ahmad A.L. (2007). Equilibrium and Kinetic Studies on Basic Dye Adsorption by Oil Palm Fibre Activated Carbon. Chem. Eng. J..

[41] De Araujo T.P., Tavares F.O., Vareschini D.T., Barros M.A.S.D. (2021). Biosorption mechanisms of cationic and anionic dyes in a low-cost residue from brewer’s spent grain. Environ. Technol..

[42] Parlayici S., Pehlivan E. (2021). Biosorption of methylene blue and malachite green on biodegradable magnetic *Cortaderia selloana* flower spikes: Modeling and equilibrium study. Int. J. Phytoremediation.

[43] Georgin J., Marques B.S., Peres E.C., Allasia D., Dotto G.L. (2018). Biosorption of cationic dyes by Para chestnut husk (*Bertholletia excelsa*). Water Sci. Technol..

[44] Tabaraki R., Sadeghinejad N. (2017). Biosorption of six basic and acidic dyes on brown alga *Sargassum ilicifolium*: Optimization, kinetic and isotherm studies. Water Sci. Technol..

[45] Gurses A., Doğar Ç., Yalçin M., Açıkyıldız M., Bayrak R., Karaca S. (2006). The Adsorption Kinetics of the Cationic Dye, Methylene Blue, onto Clay. J. Hazard. Mater..

[46] Malarvizhi R., Ho Y.-S. (2010). The influence of pH and the structure of the dye molecules on adsorption isotherm modeling using activated carbon. Desalination.

[47] Işikver Y. (2017). Removal of some cationic dyes from aqueous solution by acrylamide- or 2-hydroxyethyl methacrylate-based copolymeric hydrogels. Fibers Polym..

[48] Caparkaya D., Cavas L. (2008). Biosorption of Methylene Blue by a Brown Alga *Cystoseira barbatula* Kutzing. Acta Chim. Slov..

[49] Gurusamy A., Juang R.-S., Lee D.-J. (2002). Use of Cellulose-Based Wastes for Adsorption of Dyes from Aqueous Solutions. J. Hazard. Mater..

[50] Bhattacharyya K., Sharma A. (2005). Kinetics and Thermodynamics of Methylene Blue Adsorption on Neem (*Azadirachta indica*) Leaf Powder. Dye. Pigment..

[51] Sharma Y.C., Upadhyay S. (2009). Removal of a Cationic Dye from Wastewaters by Adsorption on Activated Carbon Developed from Coconut Coir. Energy Fuels.

[52] El-Sikaily A., Khaled A., El Nemr A., Abdelwahab O. (2006). Removal of Methylene Blue from aqueous solution by marine green alga *Ulva lactuca*. J. Chem. Ecol..

[53] Rehman M.S.U., Kim I., Han J.-I. (2012). Adsorption of methylene blue dye from aqueous solution by sugar extracted spent rice biomass. Carbohydr. Polym..

[54] Kumar V., Ramamurthi V., Sivanesan S. (2005). Modeling the Mechanism Involved during the Sorption of Methylene Blue onto Fly Ash. J. Colloid Interface Sci..

[55] Chakrabarti S., Dutta B. (2005). On the adsorption and diffusion of Methylene Blue in glass fibers. J. Colloid Interface Sci..

[56] Danouche M., El Ghachtouli N., El Arroussi H. (2021). Phycoremediation mechanisms of heavy metals using living green microalgae: Physicochemical and molecular approaches for enhancing selectivity and removal capacity. Heliyon.

[57] Yu R., Chai H., Yu Z., Wu X., Liu Y., Shen L., Li J., Ye J., Liu D., Ma T. (2020). Behavior and Mechanism of Cesium Biosorption from Aqueous Solution by Living *Synechococcus* PCC7002. Microorganisms.

